# The K167I variant of DNA polymerase β that is found in Esophageal Carcinoma patients impairs polymerase activity and BER

**DOI:** 10.1038/srep15986

**Published:** 2015-11-03

**Authors:** Yuanyuan Wang, Wenqiao Zang, Yuwen Du, Xiaonan Chen, Guoqiang Zhao

**Affiliations:** 1 College of Basic Medical Sciences, Zhengzhou University, Zhengzhou 450001, China

## Abstract

DNA polymerase β (pol β) is a key enzyme in DNA base excision repair, and an important factor for maintaining genomic integrity and stability. Esophageal carcinoma (EC) patients who have been identified as carrying the K167I variant of pol β have been shown to have decreased life expectancy. However, it is unknown if the variant affects pol β’s functions and/or how it contributes to the initiation and progression of cancer. In this study, we expressed and purified the K167I variant. Moreover, we found that K167I significantly reduced polymerase activity. As a result, the K167I substitution reduced base excision repair (BER) efficiency when assayed in a reconstitution assay or when using cellular extracts. Finally, we observed EC cells expressing the K167I variant to be sensitive to DNA damaging agents. These results suggest the K167I variant affected pol β biochemical activity resulting in impaired BER function, which might subsequently contribute to genomic instability and cancer development.

Tumorigenesis is an evolutionary process that selects for genetic and epigenetic changes, allowing evasion of anti-proliferative and cell death mechanisms that normally limit clonal expansion of somatic cells^1^. Genomic instability, a common feature in cancer cells, fuels the accumulation of oncogenic mutations, while radiation and diverse genotoxic agents remain important, and is found to exist in most tumors^2^,^3^. The DNA damage response, the guardian of genomic integrity, repairs DNA damage and is as an oncogene inducible biological barrier against the progression of cancer beyond its early stages^4^.

DNA pol β is the primary polymerase involved in base excision repair (BER), through its bifunctional deoxyribose phosphate lyase and polymerase activities, and it functions in all the sub-pathways of BER^5^,^6^,^7^,^8^. BER is a major DNA repair pathway in eukaryotic cells, it is responsible for resolving up to 20,000 lesions per cell, per day, these include oxidative and alkylation damage^9^,^10^. Of all the BER proteins identified, pol β has been demonstrated to be a key player in both short-patch BER (SP-BER) and long-patch BER (LP-BER) pathways^11^,^12^,^13^,^14^,^15^. The pol β is a 39 kDa protein that contains two domains; a dRP lyase domain (8 kDa) and a polymerase domain (31 kDa). These two domains correspond to the dRP lyase and polymerase activities which are responsible for the removal of the sugar phosphate group and the incorporation of new deoxyribonucleotides, respectively^16^.

Mutations that affect the dRP lyase or polymerase activity^17^,^18^,^19^,^20^ of pol β have been reported to impair BER efficiency and induce hypersensitive to alkylating or oxidative agents, including methyl methanesulfonate (MMS)^21^,^22^, in cells. Polymorphisms can result in biochemical alternations, BER deficiency, and predisposition to cancers^18^,^23^,^24^,^25^,^26^,^27^, therefore it is of interest to determine if and how a given polymorphism increases the likelihood of cancer occurrences. In our previous research on EC, the single nucleotide substitution that results in the K167I variant was found. The EC patients who were identified as having the K167I variant also had reduced life expectancy^28^. However, it is unknown if the variant affects pol β functions and/or how it contributes to the development and progression of cancer.

In this current study, we expressed and purified the K167I variant and found it to significantly reduced polymerase activity. Here, the K167I substitution reduced BER efficiency when assayed in a reconstitution assay and in cellular extracts. Furthermore, EC cells expressing the K167I variant were sensitive to DNA damaging agents. These results suggest that the K167I variant affected pol β biochemical activity that resulted in defective BER, which may ultimately contribute to cancer development.

## Results

### The K167I variant is defective in polymerase activity

**Figure 1:**
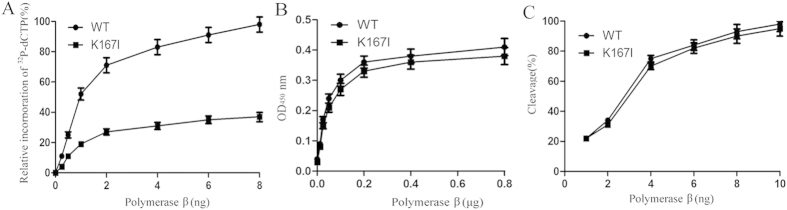
The pol β variant K167I is defective in polymerase activity. (**A**) Polymerase activity assays were performed with biotin labeled 1-nt gapped DNA substrate (pol-GAP). Separated DNA polymerization products were pulled down using Sepharose-avidin beads. After washing, the amount of radio labeled nucleotides incorporated into the products was determined by liquid scintillation counting. We found that the K167I variant (0.25 ng: 5.2 ± 0.57, 0.5 ng: 11.3 ± 1.25, 1 ng: 18.5 ± 2.31, 2 ng: 26.2 ± 2.94, 4 ng: 32.1 ± 3.51, 6 ng: 35.7 ± 3.97, 8 ng: 37.8 ± 4.84) had about 30% primer extension activity compared to the WT enzyme (0.25 ng: 11.2 ± 1.52, 0.5 ng: 25.4 ± 3.45, 1 ng: 52.6 ± 5.79, 2 ng: 71.8 ± 7.04, 4 ng: 84.5 ± 7.95, 6 ng: 91.3 ± 8.89, 8 ng: 99.1 ± 8.97). (**B**) ELISA based isotherm adsorption assays of the DNA bindi ng affinity of K167I (0.0125 μg: 0.08 ± 0.005, 0.025 μg: 0.15 ± 0.011, 0.05 μg: 0.21 ± 0.015, 0.1 μg: 0.27 ± 0.021, 0.2 μg: 0.33 ± 0.024, 0.4 μg: 0.36 ± 0.023, 0.8 μg: 0.38 ± 0.028) and WT pol β (0.0125 μg: 0.09 ± 0.005, 0.025 μg: 0.17 ± 0.010, 0.05 μg: 0.24 ± 0.015, 0.1 μg: 0.31 ± 0.021, 0.2 μg: 0.36 ± 0.020, 0.4 μg: 0.38 ± 0.023, 0.8 μg: 0.41 ± 0.028). The DNA substrate was biotin labeled pol-GAP. (**C**) Quantification of the 5′dRP lyase activity in K167I (1 ng: 22.4 ± 2.79, 2 ng: 31.7 ± 3.04, 4 ng: 70.6 ± 7.15, 6 ng: 82.1 ± 7.92, 8 ng: 89.7 ± 8.25, 10 ng: 95.4 ± 8.74) and WT pol β (1 ng: 21.9 ± 2.31, 2 ng: 35.2 ± 2.94, 4 ng: 76.2 ± 6.89, 6 ng: 84.3 ± 7.64, 8 ng: 93.1 ± 8.73, 10 ng: 98.7 ± 8.81) were shown.

To determine the effects of the K167I variant on pol β biochemical activities and biological functions, we first expressed and purified WT and K167I human pol β, then conducted primer extension experiments, and finally performed dRP lyase and DNA-binding activity assays^29^. In the primer extension assay, we found the K167I variant had approximately 70% reduction in primer extension activity compared to the WT enzyme (Fig. 1A). However, there were no differences between the K167I variant and the WT enzyme in DNA-binding activity or dRP lyase activity (Fig. 1B,C). These results were not unexpected given the K167I mutation occurs in the 31-kDa Palm domain, which is the polymerase catalytic domain.

### The K167I variant has lower BER efficiency

**Figure 2:**
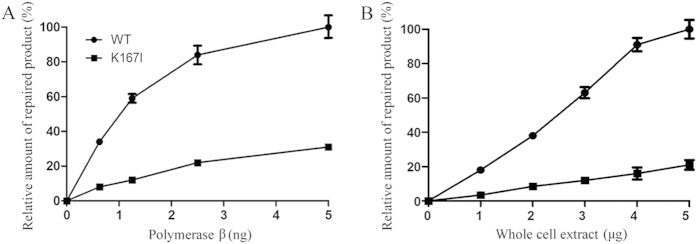
K167I significantly reduces BER efficiency. (**A**) BER reconstitution assays with purified WT and K167I pol β. The relative percentage of repaired product obtained from WT (0.625 ng: 34 ± 0.9, 1.25 ng: 59 ± 2.5, 2.5 ng: 84 ± 5.4, 5 ng: 100 ± 6.3) and K167I (0.625 ng: 8 ± 0.5, 1.25 ng: 12 ± 0.9, 2.5 ng: 22 ± 1.3, 5 ng: 31 ± 1.5) pol β samples shown. (**B**) BER reconstitution assays using whole cell extract WT and K167I. The relative percentage of repaired product obtained at different enzyme concentrations from WT (1 μg: 18 ± 0.9, 2 μg: 38 ± 1.7, 3 μg: 63 ± 3.2, 4 μg: 91 ± 3.9, 5 μg: 100 ± 5.4) and K167I (1 μg: 3 ± 0.5, 2 μg: 8 ± 1.1, 3 μg: 12 ± 1.5, 4 μg: 16 ± 3.5, 5 μg: 21 ± 2.8) pol β samples are shown.

The impaired polymerase activity of the K167I variant suggested that K167I likely affected BER function. To test this hypothesis, a BER assay using a uracil containing substrate (pol β-U)^29^ was conducted. Cleavage of the uracil lesion by the concerted action of UDG and APE1 resulted in a nicked DNA duplex, followed by the incorporation of ^32^P-dCTP, and other deoxynucleotides, which produced 20–30 nt non-ligated intermediates that were visible on a PhosphorImager. The results from this assay showed the uracil lesions were efficiently repaired in the presence of WT pol β, and resulted in a 40 nt band, but not in the presence of the K167I variant (Fig. 2A). To validate the K167I variant impacts BER efficiency, we expressed human WT and K167I pol β in pol β knockout EC9706 cells. Then nuclear extracts (NEs) from these cell lines were used in BER efficiency assays. The results from these experiments showed the NE of cells expressing WT pol β efficiently repaired the uracil lesion, and generated a fully repaired product of 40 nt, whereas the repair efficiency of the NE from cells expressing K167I pol β had approximately 80% reduction in efficient compared to the WT pol β efficiency (Fig. 2B).

### *Pol β* knockout cells expressing K167I variant demonstrate lower DNA repair capacity

**Figure 3:**
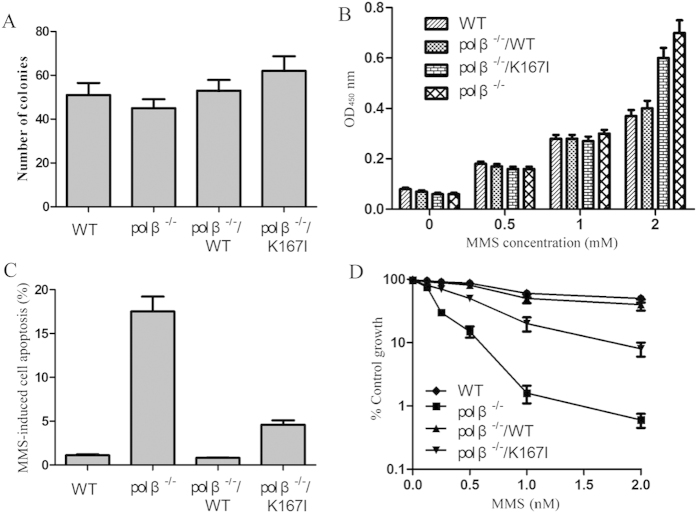
Cells containing variant K167I were sensitive to DNA damage. (**A**) Anchorage -independent growth was measured by colony formation in soft agar. Colonies with >50 cells were scored. (**B**) DNA damage assay. Cells were treated with MMS. The amount of damaged DNA lesions was detected using ARP. (**C**) MMS induced apoptosis was analyzed by flow cytometry. (**D**) Cellular sensitivity was determined by growth inhibition experiments after cells were treated with MMS.

First, we conducted colony formation assays to determine the effect of K167I on the anchorage-independent growth capacity of EC9706 cells. The EC9706 cells, WT (WT), *pol β* knockout cells (*pol β*^−/−^), and *pol β*^−/−^ cells expressing human WT or K167I pol β (*pol β*^−/−^/WT, and *pol β*^−/−^/K167I, respectively), were assayed. The results showed the colony numbers of *pol β*^−/−^/K167I was higher than that of *pol β*^−/−^/WT, however there was no statistically significant difference between the two groups (Fig. 3A). Given our observation that K167I pol β caused impaired BER we proposed the cells expressing the K167I variant would be sensitive to DNA base damaging agents. To test this hypothesis, the four cell types of EC9706 were treated with methyl methanesulfonate-sensitive (MMS). These results showed a deletion of *pol β* caused cells to accumulate DNA damage at MMS concentrations >1 mM (Fig. 3B) FACS data revealed the number of apoptotic cells from *pol β*^−/−^ or *pol β*^−/−^/K167I samples were considerably higher than in the WT group. Additionally, there was a 6-fold increase in MMS induced apoptosis in K167I cells compared to WT cells (4.6% versus 0.8%) (Fig. 3C). Data from growth inhibition assays showed MMS consistently had a stronger inhibitory effect on *pol β*^−/−^/K167I cell growth compared to WT or *pol β*^−/−^/WT cells. Following MMS treatment, the ratio of MMS treated/untreated *pol β*^−/−^/K167I cells was <10%, whereas WT or *pol β*^−/−^/WT cells was >80% (Fig. 3D).

## Discussion

Pol β is the primary polymerase involved in BER, through its bifunctional deoxyribose phosphate lyase and polymerase activities, and it is an important factor for maintaining genomic integrity and stability. Pol β fills in a single nucleotide gap and catalyzes removal of the dRP group^13^,^31^,^32^. Approximately 30% of human tumors examined for mutations in pol β appear to express pol β variants^30^. Many of these variants result from a single amino acid substitution. In this study, we expressed and purified the K167I pol β variant. Studies performed using this variant found the K167I variant was unable to catalyze DNA synthesis, but was still able to bind DNA and possessed dRP lyase activity at a level similar to that of WT pol β. We also discovered the K167I mutation significantly reduced polymerase activity, decreased BER efficiency, and overall DNA base damage repair capacity, both *in vitro* and *in vivo*. Consistent with our previous finding of EC patients who carry the K167I have reduced life expectancies, the results of anchorage-independent growth assays showed the colony number of *pol β*^−/−^/K167I was higher than that of *pol β*^−/−^/WT. However, no statistical significance was observed between these two groups. These results suggest that an individual with the K167I variant may be sensitive to endogenous and exogenous DNA damaging agents due to impaired polymerase activity and cellular base excision repair capacity.

The amino acid residue Lys167 is critical to pol β functions^33^,^34^. According to the crystal structure, K167I maps to the polymerase catalytic domain of pol β^16^. The substitution of K167I might disrupt the formation of hydrogen bonds and thus impair polymerase activity. Consistent with this hypothesis, in these experiments, K167I had approximately 30% of the DNA polymerase activity of WT pol β (Fig. 1A,B). Here, we demonstrated that K167I was unable to catalyze DNA synthesis mediated by pol β. When WT pol β binds to DNA, it catalyzes DNA synthesis and the removal of a dRP moiety, thus preparing DNA for ligation by DNA ligase III α. If K167I binds to DNA, it is unable to fill in the single nucleotide gap. Some of the unfilled gaps could lead to cell death, this is consistent with our demonstration that expression of K167I in the non-presence of WT pol β in cells sensitizes them to MMS. Exposure to MMS induced cell death in cells expressing K167I. It is likely that unfilled gaps lead to cytotoxicity, resulting in MMS sensitivity.

In the current study, we illustrated that one single nucleotide substitution can have profound consequences on protein function. The effect of an individual polymorphism is subtle, but combinations of relevant polymorphisms may additively contribute to an increased risk of human diseases^35^. Because DNA continuously undergoes alterations, either spontaneously or induced by exogenous factors, highly efficient DNA repair systems are critical to remove damaged DNA lesions and to maintain genome integrity. Genomic instability, a common feature in cancer cells, could fuel the accumulation of oncogenic mutations. In this case, the K167I pol β variant may significantly contribute to the development of human cancers through impaired polymerase activity.

## Methods

### Preparation of recombinant wild type and variant pol β

The wild type (WT) and K167I variant human pol β were obtained in our previous research^28^,^36^. WT pol β and K167I genes were inserted into the pET28a and pcDNA3.1 vectors.

### Protein expression and purification

WT or K167I pol β fusion proteins were expressed in BL21 DE3 cells. Cultures were grown at 37 °C to mid-log phase and then induced with 1mM IPTG for 4 hours. Cells were harvested by centrifugation. The enzymes (WT and K167I pol β) were purified using HisTrap FF crude Kit (GE Healthcare, Piscataway, NJ) according to the manufacturer instructions. Purification efficiency was determined by Coomassie Blue-staining of samples separated in a SDS-PAGE gel. Protein levels were quantified by Bradford protein assay (Beyotime).

### *In vitro* primer extension assay

Primer extension reactions were conducted in a solution containing (α-^32^P) dCTP, biotin labeled DNA substrate pol- GAP and pol β (WT or K167I), as previously described^29^. A portion of the reaction product was incubated with avidin-Sepharose 4B beads, washed and quantified using a liquid scintillation analyzer. The reaction product was stopped. Samples were separated in a 20% polyacrylamide gel electrophoresis (PAGE) containing 8M urea and visualized using a PhosphorImager (Molecular Dynamics, Inc).

### DNA binding assay

Biotin labeled pol-GAP DNA substrate was immobilized on a streptavidin coated 96-well Enzyme Linked ImmunoSorbent Assay (ELISA) plate and washed three times with binding buffer. This was followed by the addition of 0–0.8 mg WT or K167I pol β, and samples were then incubated overnight at 4 °C. Next, samples were incubated with a mouse anti-pol β antibody (Santa Cruz Biotech) and a goat anti-mouse IgG/HRP antibody (Santa Cruz Biotech). Samples were then treated with tetramethyl benzidine (TMB) and the reactions were stopped using HCl. The optical density (OD) was measured at a wavelength of 450 nm (OD_450_) on a microplate reader.

### 5′dRP lyase assay

The 5′dRP-primer substrate pol β-U^29^ was labeled with (γ-^32^P)-ATP at the 5′-end on the U containing oligonucleotide, and followed by treatment with pol β-U substrate with uracil DNA glycosylase (UDG) and APE1. The incised APsite- containing DNA was then incubated (20 min, 37 °C) with WT or K167I pol β (0–10 ng) in buffer. Formamide containing gel loading buffer was then added and reaction products were resolved on 20% polyacrylamide gels containing 8M urea and visualized using a PhosphorImager.

### Reconstituted base excision repair assay

A 5′-end-labeled pol β U substrate was used as a BER substrate. Complete repair reactions were carried out in 20 μl reaction mixtures. The UDG treated substrate (20 nM) was incubated for 5 min with APE1, purified pol β (WT or K167I), and T4 DNA ligase in buffer, at 37 °C, for 30 min. The reaction products were resolved on a 20% denaturing polyacrylamide gel and visualization using a PhosphorImager.

### Cell lines and culture

Our lab had previously established WT and *pol β* null (*pol β*^−/−^) EC9706 cells lines. Cells were maintained in RPMI 1640 medium supplemented with 10% fetal bovine serum (FBS; Gibco BRL, Gaithersburg, MD, USA), and incubated at 37 °C, and 5% CO_2_. Both *pol β*^−/−^/WT and *pol β*^−/−^/K167I stable cell lines were generated using pcDNA3.1-WT/K167I vectors and Lipofectamine™2000 (Invitrogen) according to the manufacturer’s instructions.

### Colony formation assay

The four EC9706 cell lines (WT, pol β^−/−^, pol β^−/−^/WT, and pol β^−/−^/K167I) were washed, trypsinized, counted, and suspended (1000 cells per dish) in 2 ml complete medium plus 0.4% agar (Sigma, St Louis, MI). The agar-cell mixture was plated on top of a bottom layer consisting of 1% agar in complete medium. Colony formations, which occurred in approximately two weeks, were observed. The newly formed colonies were fixed with ethanol, stained with crystal violet, and colonies with >50 cells were scored.

### Preparation of cell extract

Cells were cultured and incubated overnight to reach a mid-exponential growth phase, washed three times with PBS and resuspended at 10^6^ cells/20 μl in Buffer I. After the addition of an equal volume of Buffer II, the cell suspension was rocked for 1 h at 4 °C, and centrifuged; the supernatant was recovered, and stored at −80 °C. Protein concentrations were determined by Bradford protein assay^37^.

### DNA damage assay

Briefly, a monolayer of cells was treated with MMS for 1 h during the logarithmic growth phase. Cells were then washed, trypsinized and collected. Genomic DNA was extracted using DNAzol reagent (Invitrogen) and coated onto ELISA plates and detected using an AP-site specific reagent, Aldehyde-Reactive Probe (ARP) (Invitrogen), samples were incubated with HRP-conjugated streptavidin and TMB. Reactions were stopped using 1N HCl. The optical density (OD) was measured at a wavelength of 450 nm (OD_450_) on a microplate reader.

### MMS sensitivity assay

WT and *pol β* null (*pol β*^−/−^) EC9706 cells were seeded at 2000 per well and incubated overnight at 37 °C. Samples were treated with serial dilutions of MMS for 1 h, then washed and incubated in fresh medium, DMEM containing 10% FBS, under normal growth conditions for 72 h. The optical density (OD) was measured at a wavelength of 490 nm (OD_490_) on a microplate reader by the CellTiter 96 AQueous one-solution cell proliferation assay kit (Promega).The number of viable cells was counted using an inverted microscope (200 × magnification). Data are expressed as the percentage of growth relative to untreated controls.

### Cell apoptosis analysis

Harvested cells treated with MMS were resuspended at a density of 1 × 10^6^ cells/mL in 1 × binding buffer. After double staining with FITC-Annexin V and propidium iodide (PI) using the FITC Annexin V Apoptosis Detection Kit I (BestBio, Shanghai, China), cells were analyzed using a FACScan^®^ flow cytometer (BD Biosciences) equipped with Cell Quest software (BD Biosciences).

### Statistical analysis

Statistical analysis was conducted using SPSS 17.0 software. All data are expressed as means ± standard deviation (SD). Two-tailed unpaired Student’s t test and One-way analysis of variance (ANOVA) was used to analyze data. Multiple comparison between the groups was performed using S-N-K method. Results were considered significant when *P*-values were <0.05.

## Additional Information

**How to cite this article**: Wang, Y. *et al.* The K167I variant of DNA polymerase β that is found in Esophageal Carcinoma patients impairs polymerase activity and BER. *Sci. Rep.* **5**, 15986; doi: 10.1038/srep15986 (2015).
